# The never-ending story: from pluripotency to plant developmental plasticity

**DOI:** 10.1242/dev.117614

**Published:** 2015-07-01

**Authors:** Christophe Gaillochet, Jan U. Lohmann

**Affiliations:** Department of Stem Cell Biology, Centre for Organismal Studies, University of Heidelberg, Heidelberg, 69120, Germany

**Keywords:** Plant development, Pluripotency, Stem cells

## Abstract

Plants are sessile organisms, some of which can live for over a thousand years. Unlike most animals, plants employ a post-embryonic mode of development driven by the continuous activity of pluripotent stem cells. Consequently, plants are able to initiate new organs over extended periods of time, and many species can readily replace lost body structures by *de novo* organogenesis. Classical studies have also shown that plant tissues have a remarkable capacity to undergo de-differentiation and proliferation *in vitro*, highlighting the fact that plant cell fate is highly plastic. This suggests that the mechanisms regulating fate transitions must be continuously active in most plant cells and that the control of cellular pluripotency lies at the core of diverse developmental programs. Here, we review how pluripotency is established in plant stem cell systems, how it is maintained during development and growth and re-initiated during regeneration, and how these mechanisms eventually contribute to the amazing developmental plasticity of plants.

## Abstract (precis)

**Summary:** This Review discusses how pluripotency is established in plant stem cell systems, how it is maintained during development and growth, and how it is re-initiated during regeneration.

## Introduction

Over the past few decades, significant progress has been made in deciphering the regulatory mechanisms that control the development of complex multicellular organisms – an intricate process starting with the fusion of the male and female gametes, which form the zygote. This totipotent cell displays the remarkable capacity to self-renew and, after a series of cell divisions and cellular fate transitions, to give rise to all the cell types composing the adult organism. Further along the course of ontogenesis, populations of adult pluri- or multipotent stem cells are set aside, which allow the establishment, homeostasis and regeneration of tissues. Despite multicellularity arising independently in plants and animals, this basic developmental scheme is conserved between the two kingdoms. In line with this, common regulatory mechanisms controlling stem cell activities in a wide range of model organisms as well as among different stem cell niches within the same organism begin to emerge (Heidstra and Sabatini, 2014; Lodha et al., 2009; Simons and Clevers, 2011). It has turned out that plant stem cell niches are not only similar to their animal counterparts at the level of their overall design, but also share many regulatory principles, highlighting the constraints in stem cell system evolution. Against this backdrop, we review the control of pluripotency in plants, covering stem cell systems, signalling and the contribution of the cell cycle machinery, as well as important insights from *in vitro* experiments. To conclude, we discuss how the mechanisms controlling pluripotency might underlie the extraordinary developmental plasticity observed in plants.

## Meristems: the home of plant stem cells

The life-long control of cellular pluripotency is a key process during plant development, as, in contrast to most animals, the bulk of the plant body is generated after embryogenesis. Classical work on *in vitro* plant tissue cultures revealed substantial cell fate plasticity and the basis for the regenerative capacity of plants (Skoog and Miller, 1957). By cultivating small pieces of tobacco leaves under defined conditions, proliferating cells with totipotent properties, termed callus, were induced, and fully functional adult plants could be generated from this tissue (Vasil and Hildebrandt, 1965a,b). The discovery of these intriguing properties of plant tissues raised a number of pressing questions: which cells maintain pluripotency *in vivo*, how is pluripotency controlled at the genetic and molecular level, and how can pluripotency be re-established from adult somatic tissues?

**Fig. 1.**
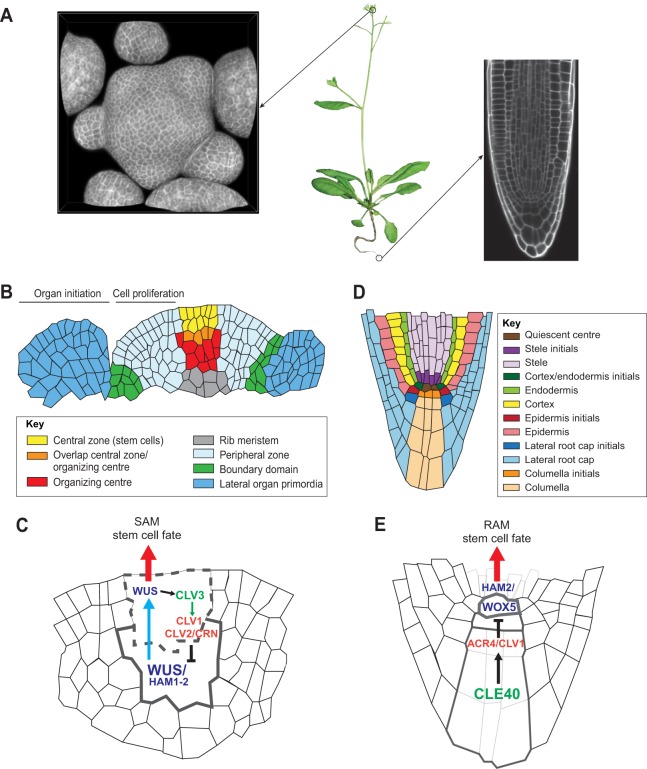
**Plant meristems: tissue structure, cellular organization and common gene regulatory networks.** (A) *Arabidopsis thaliana* plant 25 days after germination, with a close-up view of the inflorescence shoot apical meristem (SAM, left) and the root apical meristem (RAM, right). (B) Schematic representation of a longitudinal section through the SAM, showing the different functional domains. (C) In the SAM, *WUS* is expressed in the organizing centre (OC), and WUS protein moves symplastically through plasmodesmata to the overlying domain (the central zone, CZ) to instruct stem cell fate. WUS directly or indirectly promotes the expression of *CLV3*, which encodes a small secreted peptide that signals through intracellular signal transduction pathways, involving CLV1 and CLV2/CRN to restrict *WUS* expression in the underlying cells. In the OC, HAM1 and HAM2 form a protein complex with WUS and act together to control stem cell fate. (D) Schematic representation of a longitudinal section through the RAM, illustrating the different root cell lineages. (E) In the RAM, *WOX5* is expressed specifically in the QC. WOX5 physically interacts with HAM2 to instruct distal stem cell fate non-cell-autonomously. The activity of the small peptide CLE40, which acts together with the CLV1/ACR4 receptor complex, restricts WOX5 expression. Note that a WOX-HAM/CLE genetic module functions in both the SAM and the RAM to control stem cell fate. Black arrow, gene regulation; blue arrow, intercellular protein movement; green arrow, receptor-ligand interaction; red arrow, biological process.

Plant pluripotent stem cells reside in microenvironments called meristems. Two primary meristems, the shoot apical meristem (SAM) and the root apical meristem (RAM), are responsible for plant longitudinal growth and are located at the tip of the stem and root, respectively (Fig. 1). In addition, plants develop a secondary meristem, the cambium, that allows them to grow radially, and which contributes cells to their vasculature and for mechanical support structures (Jouannet et al., 2015). The control of meristematic activity is therefore crucial for allowing plants to establish their body plan, maintain tissue homeostasis and adapt their development to fluctuating environments. As plant cells are encased within a rigid cell wall structure and are thus immobile, plant growth is mostly driven by cell division and expansion. In the shoot and root meristems, groups of mitotically active cells divide and create fields of displaced cells, which adopt different cellular functions as they transit through different functional domains (Gaillochet et al., 2015; Perilli et al., 2012). In recent years, a refined picture of the regulation of meristem activity has started to emerge, which involves the interplay between phytohormonal signals, transcriptional regulatory networks and chromatin remodelling factors. However, how this network responds to environmental signals and how these inputs are translated into cellular behaviour ultimately leading to plant developmental plasticity still remains to be elucidated.

## Meristem organization and common molecular modules controlling plant stem cells

Recent studies have begun to elucidate the organisation of the SAM and the RAM, and the key mechanisms that regulate these stem cell niches. Whereas these studies have highlighted a number of differences between plant stem cell niches, they have also revealed some key common modules as well as regulatory mechanisms that appear to be shared between plant and animals stem cells.

### SAM cellular organization and regulatory control

Shoot stem cells are the source of all aboveground tissues of a plant and are embedded in the SAM (Fig. 1B). This dome-shaped structure is organized in three clonally distinct layers: L1 and L2 cells constitute the two outermost layers and divide exclusively anticlinal, with L1 facing the environment and L2 located directly underneath. By contrast, cells of the L3 layer located below L2 divide in all orientations. Thus, individual cell layers give rise to independent cell lineages and contribute differentially to developing organs. At the centre of the meristem, stem cells divide only rarely, and part of their progeny is displaced laterally towards the peripheral zone (PZ), which exhibits a much higher cell division rate (Reddy et al., 2004). As a consequence of this division activity, cells are continuously pushed further towards the periphery, where they are eventually recruited to form the lateral organs or the vascular tissues and the stem.

Molecular studies have defined additional, distinct functional domains within the SAM (Fig. 1B). The organizing centre (OC) located basally of the stem cells acts to instruct and maintain pluripotency in the overlying stem cells of the central zone (CZ). At the molecular level, the OC is defined by expression of the homeodomain transcription factor *WUSCHEL* (*WUS*), which is required for stem cell activity (Mayer et al., 1998). WUS is the founding member of the WUS-homeobox (WOX) family, which plays important roles in diverse plant stem cell systems (Lu et al., 2015; Nardmann and Werr, 2006). Recent studies showed that symplastic movement of WUS protein from the OC to the CZ through plasmodesmata is necessary to maintain stem cell fate (Fig. 1C); degrading WUS protein in stem cells or blocking its movement results in stem cell depletion (Daum et al., 2014; Yadav et al., 2011).

The genetic regulation of shoot stem cell activity involves a negative-feedback loop, which is spatially embedded in a bi-directional intercellular communication system (Fig. 1C). WUS directly or indirectly activates stem cell expression of *CLAVATA3* (*CLV3*), which encodes a small peptide, and CLV3 in turn signals back to the OC by restricting the *WUS* expression domain (Brand et al., 2000; Ohyama et al., 2009; Schoof et al., 2000; Yadav et al., 2011).This communication requires the secretion of CLV3 into the intercellular space, where it acts through the leucine-rich repeat (LRR) receptor-like kinase (RLK) CLAVATA1 (CLV1) by directly binding to its ectodomain. In addition, CLV3 signal is also relayed through cooperative activity of CLAVATA2 (CLV2)/CORYNE (CRN) receptor protein complex and through the RECEPTOR-LIKE PROTEIN KINASE 2 (RPK2), which together delineate three parallel pathways mediating the communication from the CZ to the OC (Bleckmann et al., 2009; Clark et al., 1997; Kinoshita et al., 2010; Müller et al., 2008; Ogawa et al., 2008; Rojo et al., 2002).

The signal transduction downstream of these receptors to *WUS* regulatory regions is less clear but involves the activity of heterotrimeric GTP-binding proteins and potentially mitogen-activated protein kinase (MAPK) signalling (Betsuyaku et al., 2011; Bommert et al., 2013; Ishida et al., 2014).

In parallel to this local regulatory system that maintains stem cell identity, cells are kept in an undifferentiated state throughout the SAM by the activity of *SHOOTMERISTEMLESS* (*STM*), a member of the KNOTTED-like homeobox (KNOX) gene family, which has overlapping activities with KNOTTED1-LIKE HOMEOBOX GENE 6 (KNAT6) and BREVIPEDICELLUS (BP) (Belles-Boix et al., 2006; Byrne et al., 2002; Long et al., 1996). STM represses the activity of the differentiation genes *ASYMETRIC LEAVES 1* (*AS1*) and *AS2,* which in turn form a dimer and repress KNOX gene expression to promote cell differentiation (Byrne et al., 2002, 2000; Guo et al., 2008). Therefore, the SAM boundary is defined by a double-negative-feedback loop, which results in the differentiation of cells that are pushed out of the SAM.

### RAM cellular organization and regulatory control

At the extreme basal end of the plant, the RAM is the source for the entire underground tissues (Fig 1A). In contrast to the SAM, the cellular structure of the RAM follows a stereotypical organization (Fig. 1D), with all stem cells, also termed initial cells, surrounding an ‘organizer’ region called the quiescent centre (QC) (van den Berg et al., 1997). The QC is composed of four rarely dividing cells and is marked by *WUSCHEL-RELATED HOMEOBOX 5* (*WOX5*) activity (Fig. 1E), which is essential to maintain stem cell fate non-cell autonomously (Bennett et al., 2014; Forzani et al., 2014; Sarkar et al., 2007). The adjacent stem cells give rise to distinct cell lineages through stereotypical asymmetric cell divisions, during which stem cells remain in direct proximity to the QC. Similar to the SAM, the control of distal stem cell fate involves intercellular communication between two functional domains but, in this case, differentiating columella cells counterbalance stem cell activity by producing CLAVATA3/ESR-RELATED 40 (CLE40) peptide (Fig. 1E). At the molecular level, CLE40 positively regulates the abundance of the receptor protein complex CRINKLY4 (ACR4)/CLV1, to restrict *WOX5* expression and thereby keeps the number of columella initials in balance (Stahl et al., 2013, 2009).

### Common regulatory modules in plant stem cell niches

From research on diverse plant stem cell niches, it has emerged that the WOX/CLE module is unifying in controlling stem cell activity via inter-domain cross-communication. Strikingly, WUS and WOX5 functions are interchangeable *in vivo* and the stem cell defects of *wus* or *wox5* mutants can largely be rescued by promoter swap experiments (Sarkar et al., 2007). Furthermore, shoot and root stem cell systems rely on the activity of CLE peptides, which signal through receptors belonging to the LRR RLK family. In particular, CLV1 mediates a signal between the stem cells and their niche in both the SAM and the RAM (Fig. 1C,E) (Ogawa et al., 2008; Stahl et al., 2013). In addition to acting in primary meristems, the WOX/CLE signalling module controls stem cell activity in the cambium. However, in this context, CLE41 and CLE44 peptides signal through the LRR RLK TDIF RECEPTOR (TDR) to stimulate cambial cell activity and to repress xylem differentiation by promoting *WUSCHEL-RELATED HOMEOBOX 4* (*WOX4*) expression (Etchells and Turner, 2010; Hirakawa et al., 2010, 2008). The shared molecular mechanisms of plant stem cell systems even extend to the level of essential co-factors, as HAIRY MERISTEM (HAM) proteins can form protein complexes with WUS, WOX5 and WOX4 and are required to promote shoot, root and cambial stem cell activity, respectively (Fig. 1C,E) (Zhou et al., 2015). This clear conservation of the regulatory building blocks controlling stemness raises interesting questions regarding the evolutionary origin and trajectory of plant stem cell systems. Despite the intriguing similarities of the actors involved, it is important to note that the wiring between the different components diverges substantially between the diverse stem cell systems and involves different spatial as well as logical organizations (Fig. 1C,E).

### Conserved mechanism for stem cell control in plants and animals

Whereas the molecular players underlying stem cell niche organization diverge between plants and animals, other mechanisms controlling stem cell activity seem to be conserved even between the kingdoms of life. In both animals and plants, proteins of the Polycomb (PcG) and the Trithorax (trxG) families play opposing roles by promoting or restricting chromatin accessibility at gene regulatory regions, respectively. Typically, Polycomb Repressive Complex 2 (PRC2) promotes H3K27 and H3K9 methylation, leading to recruitment of the PRC1 complex, which ultimately promotes chromatin compaction and stable gene repression (Margueron and Reinberg, 2011). However, the trxG complexes catalyses H3K4 and H3K36 methylation and is generally associated with gene activation. Within plant meristems, these complexes play key roles in regulating cell identity and cell fate progression by controlling chromatin accessibility, which subsequently directly or indirectly influences the expression of key transcription factors (Bratzel et al., 2010; Lodha et al., 2013; Schubert et al., 2006). Strikingly, removing the activity of redundant PRC2 factors leads to massive overproliferation and a failure of cell differentiation, resulting in masses of undifferentiated cells (Schubert et al., 2005).

Another example is transcriptional elongation, which has been assigned a crucial role in cell differentiation in animal stem cell systems (Di Micco et al., 2014; Hanyu-Nakamura et al., 2008; Park et al., 2013; Zhang et al., 2003). Interestingly, interfering with the function of the transcriptional elongation factor MINIYO (IYO) in *Arabidopsis* leads to the development of a larger shoot stem cell system together with an additional cell layer of columella stem cells and multiple files of procambium in the root, collectively suggesting a delay in the onset of cell differentiation (Sanmartín et al., 2011). The amplification of certain cell types in these mutants can be related to the phenotypes observed in mice expressing increased levels of the oncogene c-MYC, which causes transcriptional amplification of pre-existing transcriptional programs rather than targeting specific genes, resulting in a variety of tumorous cell types (Lin et al., 2012).

With more details on plant stem cell control emerging, it will be exciting to see whether additional shared molecular mechanisms will be identified.

## Phytohormonal cross-talk controlling stem cell activity

Early studies on plant tissue culture and regeneration from callus defined crucial roles for the plant hormones auxin and cytokinin in coordinating cell divisions and cell differentiation (Skoog and Miller, 1957). Almost 60 years after these discoveries, our understanding of these hormonal pathways suggests that they are in fact involved in many developmental processes, ranging from setting up the plant body plan to responding to environmental stresses (Kieber and Schaller, 2014; Vanneste and Friml, 2009). Accordingly, maintaining the balance between auxin and cytokinin is a key feature of plant stem cell regulation, both to maintain stem cell activity and to define the functional domains guiding stem cell differentiation. Phytohormones also play a key role in providing positional information within meristems [reviewed in more detail by Gaillochet et al. (2015); Perilli et al. (2012)].

### Cytokinin and shoot stem cell activity

**Fig. 2.**
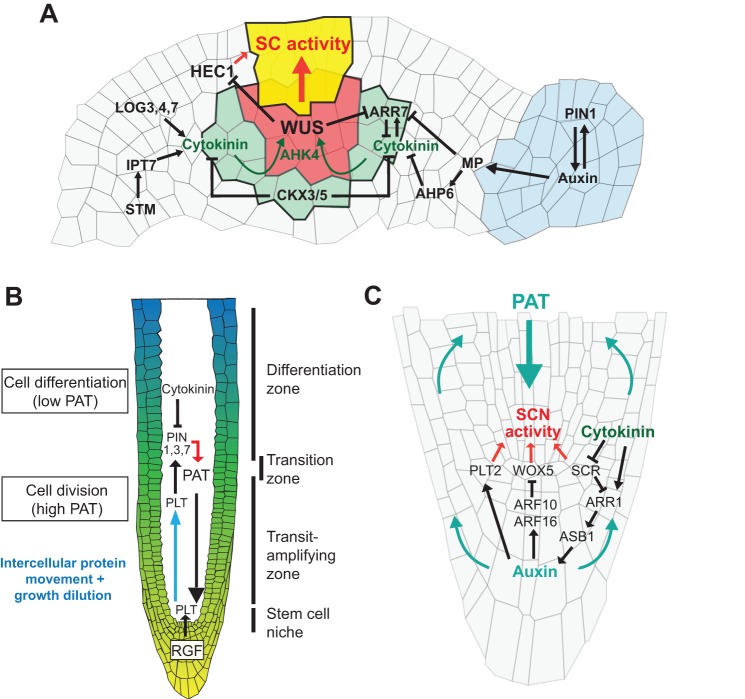
**Auxin-cytokinin cross-talk in meristems.** (A) In the OC (red), *WUS* integrates cytokinin signalling (green+red) and controls stem cell (SC) activity together with HEC1. Cytokinin biosynthesis depends on two groups of enzymes, which are encoded by the LOG (*LOG3*, *LOG4*, *LOG7*) and IPT (*IPT7*) gene families. STM regulates cytokinin production by activating *IPT7* expression, whereas cytokinin degradation is catalysed by CKX3 and CKX5. WUS facilitates cytokinin signalling by directly repressing the expression of type-A ARRs (*ARR7*). Auxin also modulates stem cell activity via regulation of type-A ARRs by the ARF transcription factor MP. In addition, it is responsible for determining the sites of primordia initiation, which involves PIN1, MP and AHP6. (B) The RAM is composed of different functional domains. Basally, formative stem cell divisions in the stem cell niche give rise to different cell lineages and, as the root grows, cells transit successively through a transit-amplifying zone, a transition zone and, finally, a differentiation zone in which they start elongating. The balance between cytokinin and auxin positions the transition zone and therefore controls progenitor cell entry into differentiation. Polar auxin transport (PAT) is regulated by PIN family auxin transporters. Auxin transport and signalling are tightly connected to the activity of PLT transcription factors. Cytokinin promotes cell entry into differentiation by dampening the accumulation of PIN1, PIN3 and PIN7. (C) Stem cell niche activity (SCN) in the root involves the concerted activity of *PLT2*, *WOX5* and *SCR*. These genes are integrated in the hormonal transcriptional network and modulate the auxin-cytokinin balance. In addition to promoting stem cell fate through the positive regulation of PLT genes, auxin promotes distal stem cell differentiation by repressing *WOX5* expression through *ARF10* and *ARF16*. Cytokinin modulates auxin biosynthesis by repressing *SCR* and by signalling through the type-B ARR ARR1, which subsequently promotes auxin biosynthesis through its positive effect on *ASB1* expression.

In the SAM, phytohormonal pathways are integrated into local transcriptional networks and control the level of *WUS* expression, which subsequently regulates shoot stem cell activity. The cytokinin signalling domain encompasses the OC and the neighbouring cell layers, where cytokinin promotes *WUS* expression (Fig. 2A) (Buechel et al., 2010; Gordon et al., 2009; Müller and Sheen, 2008; Zürcher et al., 2013). Importantly, WUS sensitizes the OC to cytokinin by directly repressing the expression of type-A *ARABIDOPSIS RESPONSE REGULATORS* (*ARRs*), which are negative regulators of cytokinin signalling, and thereby promotes its own expression (Gordon et al., 2009; Leibfried et al., 2005). Cytokinin levels in the SAM are modulated by the activities of both biosynthetic and catabolic enzymes (Kieber and Schaller, 2014). Among them, the LONELY GUY (LOG) cytokinin riboside 5′-monophosphate phosphoribohydrolases produce active cytokinin, and loss of LOG activity in rice results in severe meristem defects (Kurakawa et al., 2007; Kuroha et al., 2009). Furthermore, *ISOPENTENYL-TRANSFERASE* (*IPT*) enzymes are required for maintaining SAM cell proliferation potential downstream of STM, which promotes the expression of the gene coding for IPT7 (Jasinski et al., 2005; Yanai et al., 2005). However, cytokinin levels are limited by the activity of catabolic enzymes, which are encoded by members of the *CYTOKININ OXYDASE/DEHYDROGENASE* (*CKX*) gene family, and the reduction of CKX3 and CKX5 function consequently results in the formation of larger SAMs with a broader *WUS* expression domain (Bartrina et al., 2011).

### Auxin-cytokinin cross-talk in the SAM

In contrast to cytokinin, which promotes proliferation, auxin signalling guides primordium initiation (Fig. 2A), leading to the formation of lateral organs, leaves or flowers (Reinhardt et al., 2000). The regular arrangement of lateral organs along the main plant axis (phyllotaxis) is regulated mainly by a feed-forward system, involving the intercellular transport of auxin by the PIN-FORMED1 (PIN1) auxin efflux carrier and auxin concentration or flux, which in turn impinges on PIN expression and localization [de Reuille et al. (2006); Jönsson et al. (2006); Smith et al. (2006); reviewed by Traas (2013)].

In addition to its role in the dynamic specification of organ initiation sites at the periphery of the SAM, auxin synergizes with cytokinin to promote stem cell activity in the centre of the meristem, as shown in classical tissue culture experiments (Skoog and Miller, 1957). To balance organ formation with stem cell activity, auxin signals through the AUXIN RESPONSE FACTOR (ARF) transcription factor MONOPTEROS (MP), which directly represses the expression of *ARR7* and *ARR15*, thereby enhancing cytokinin signalling output and thus *WUS* expression (Zhao et al., 2010). Furthermore, MP also promotes the expression of *ARABIDOPSIS HISTIDINE PHOSPHOTRANSFER PROTEIN 6* (*AHP6*), which interferes with the cytokinin signal transduction pathway and restricts the cytokinin-signalling domain to the centre of the SAM. Accordingly, loss of *AHP6* function results in a SAM with stronger cytokinin response at the incipient primordia, which in turn impairs the precise timing of lateral organ growth (Besnard et al., 2014).

### Auxin and root stem cell niche function

In the RAM, auxin is tightly wired to a set of transcriptional regulatory pathways and exerts diverse influences on the stem cell niche. At the root tip, auxin is distributed in a gradient-like fashion with a maximal concentration at the QC brought about by PIN-dependent transport following a reflux-loop design (Fig. 2B,C) (Blilou et al., 2005; Grieneisen et al., 2007; Petersson et al., 2009; Sabatini et al., 1999). The auxin-signalling peak at the QC promotes the expression of PLETHORA (PLT) transcription factors, the activity of which in turn affects auxin distribution, as PLT transcription factors control the expression of *PIN1*, *PIN3* and *PIN7* (Fig. 2B) (Blilou et al., 2005). Interestingly, PLT2 protein distribution follows the auxin gradient and is established via intercellular protein movement and growth-dependent dilution from a narrow domain at the root tip, where auxin signalling is maximal (Mähönen et al., 2014). This graded distribution is relevant, as PLT proteins act in a dose-dependent manner, with their activity ranging from stem cell niche maintenance to specifying transit-amplifying cell identity and triggering cell differentiation (Galinha et al., 2007; Mähönen et al., 2014). PLT expression is also regulated by tyrosine-sulfated peptides encoded by the ROOT GROWTH FACTOR (RGF) gene family (Fig. 2B) that also control the expression levels of auxin biosynthesis genes and that are crucial for stem cell niche activity (Matsuzaki et al., 2010; Zhou et al., 2010). Whereas auxin contributes to establish the root stem cell niche, it also participates in columella cell differentiation at the distal part of the RAM by positively regulating the expression of *ARF10* and *ARF16*, which in turn repress *WOX5* expression, demonstrating the exquisite context-dependent activity of this phytohormone (Fig. 2B) (Ding and Friml, 2010).

### Auxin-cytokinin cross-talk in the RAM

As in the shoot, the balance between auxin and cytokinin has a key function in controlling the root stem cell niche, from its specification to its maintenance. During embryogenesis, the QC arises from an asymmetric cell division of a founder cell, the hypophysis. During this process, auxin signalling specifies distinct apical and basal cell fates and acts by cell-autonomously repressing cytokinin signalling through the promotion of *ARR7* expression. This eventually leads to the formation of an apical cell with high CK output and a basal cell with high auxin signalling and, accordingly, interfering with the establishment of this hormonal response pattern leads to root stem cell niche defects (Müller and Sheen, 2008).

In contrast to its role in the SAM, auxin is the key hormone in defining the position of the root stem cell niche, whereas cytokinin instructs cell differentiation (Ioio et al., 2008; Sabatini et al., 1999). Cytokinin dampens the expression of *SCARECROW* (*SCR*), which encodes a GRAS domain transcription factor that is necessary and sufficient to cell-autonomously instruct QC fate and is a key player relaying inputs from cytokinin and auxin signals (Moubayidin et al., 2013; Sabatini et al., 2003; Zhang et al., 2013). SCR directly represses the expression of *ARR1*, which belongs to the type-B ARR gene family. In this cellular context, ARR1 promotes auxin biosynthesis by activating the expression of *ANTHRANILATE SYNTHASE BETA SUBUNIT 1* (*ASB1*) (Fig. 2C). The modulation of this regulatory cascade also has a non-cell autonomous effect through the elevation of auxin concentration at the root tip, which results in a shift of the auxin gradient and thus changes the auxin/cytokinin balance in the differentiation zone (Moubayidin et al., 2013).

## Stem cells, the cell cycle machinery and mitotic progression

A central function of stem cell niches is to allow the controlled amplification of cell lineages originating from a small number of stem cells. As plant meristems carry sub-populations of cells, which generate the whole plant body and exhibit divergent mitotic activity, precise spatio-temporal control of the cell cycle within meristems is required. Importantly, cells undergoing differentiation in plants do not simply shut down cell cycle activity, but frequently undergo multiple cycles of endo-reduplication, making cell-cycle regulation an essential component of the developmental program throughout the life of a typical plant cell. Recent research into the mechanisms controlling cell cycle progression within meristems has not only identified important molecular players, but has also highlighted that stem cell niches can respond dynamically to internal or external cues in order to adjust their cell proliferation rate.

### Cell cycle-mediated control of shoot stem cells

The stem cells at the centre of the dome-shaped shoot meristem (the CZ) divide slowly, with division cycles ranging from 36 h to 72 h, whereas cells in the peripheral zone (PZ) divide much more rapidly, with divisions occurring every 18 h to 48 h (Reddy et al., 2004). The intriguing variability of cell cycle length within the SAM has raised the idea that individual cells respond to temporally fluctuating signals (Reddy et al., 2004). However, how transient inputs are translated to stable cell behaviour and coordinated at the population level to ensure robust SAM morphogenesis remains unclear.

The WUS/CLV3 feedback loop has been shown to play an important role in controlling cell proliferation rate in the SAM. Live imaging experiments after transient reduction of *CLV3* mRNA levels revealed that CLV3 not only constrains stem cell fate to the CZ but also restricts cell division rates in the PZ (Reddy and Meyerowitz, 2005). A similar effect on the cell cycle has been observed after elevating *WUS* levels within the CZ (Yadav et al., 2010). Together, these experiments revealed that, besides playing an instructive role in controlling stem cell identity, the *WUS/CLV3* system acts non-cell autonomously to control PZ cell cycle length, thereby coordinating stem cell fate with cell differentiation (Reddy and Meyerowitz, 2005; Yadav et al., 2010).

Recently, the bHLH transcription factor HECATE1 (HEC1) was shown to control stem cell activity within the SAM in a cell-type specific manner. Uncoupling *HEC1* expression levels from negative inputs in the CZ leads to a dramatic expansion of the shoot stem cell system independently of *CLV3* function. Strikingly, *WUS* and *CLV3* expression levels are fully suppressed once strong fasciation of these meristems has manifested, suggesting that HEC1 can largely bypass the requirement for the *WUS*/*CLV* system (Schuster et al., 2014). The mechanisms underlying the potent stem cell-promoting function of HEC1 have only begun to emerge, but analysis of HEC1-responsive genes point to a prominent role for cytokinin signalling and cell cycle regulators.

Besides its positive influence on *WUS* expression, cytokinin stimulates stem cell proliferation in the SAM by promoting the G1-S transition through the regulation of the cell cycle machinery component CYCLIN D3 (CYCD3) (Gordon et al., 2009; Riou-Khamlichi, 1999). In addition, CYCLIN-DEPENDENT KINASEs (CDK) play a key role in maintaining the integrity of the SAM, as modulating the expression of *CDKB2;1* and *CDKB2;2* strongly compromises meristem integrity and activity (Andersen et al., 2008). Another key check-point required for SAM function is the entry point into the cell cycle, and expressing a dominant negative form of CYCLIN DEPENDENT KINASE A;1 (CDKA;1) leads to premature cell differentiation (Gaamouche et al., 2010). Although some components of the cell cycle machinery have been shown to play a role in regulating SAM activity, the precise connections between the core transcriptional network, the control of cell cycle genes and the subsequent cellular behaviour remain to be uncovered. Preliminary studies quantifying various SAM parameters in diverse environmental conditions show intriguing plasticity at the level of cell cycle activity, core regulators and SAM size (Geier et al., 2008). However, the level at which environmental signals are sensed and integrated into the regulatory network of the SAM remains unknown.

### The cell cycle machinery in root stem cells

Cell cycle control is also important in the root meristem and serves diverse functions at different levels. First, stem cell maintenance at the root tip depends on asymmetric cell divisions, and their precise spatio-temporal control is required for the proper formation of specific tissue layers [reviewed by De Smet and Beeckman (2011)]. Second, cell division rates in the transit-amplifying zone dictate the number of cells entering differentiation and therefore control root growth. Finally, cells in the QC residing at the centre of the root stem cell niche exhibit a very low division activity, which is required for maintaining stem cell niche longevity.

At the proximal RAM region, cells entering the transit-amplifying zone ramp up their division rate (Fig. 2B). In addition to setting up the niche, PLT2 also controls this process, and elevated PLT2 levels in the transit-amplifying zone promote progenitor cell proliferation and repress cell elongation in the differentiation zone (Galinha et al., 2007). Interestingly, a central player in controlling root stem cell niche activity is the RETINOBLASTOMA-RELATED (RBR) protein, a homolog of the animal pRB. In animal cells, pRB physically interacts with the E2F-DP protein complex and prevents G1-S cell cycle progression. Upon hyperphosphorylation by CDKs, it is released from the E2F-DP complex, which can then transcriptionally activate S phase genes [reviewed by Weinberg (1995)]. In the root, loss of RBR function leads to supernumerary columella stem cells, which do not result from a higher columella stem cell division rate but instead arise due to their inability to differentiate. Consistently, proximal stem cell division rate is not affected (Wildwater et al., 2005). As in animal systems, RBR forms a protein complex with the cell cycle regulator E2Fa and modulates the expression of diverse downstream cell cycle regulators (Magyar et al., 2012). Furthermore, KIP-RELATED PROTEIN 2 (KRP2), CYCD3 and E2Fa also influence columella stem cell progression to differentiation, in line with their physical interaction with RBR (Magyar et al., 2012; Wildwater et al., 2005).

### A molecular framework for quiescence

**Fig. 3.**
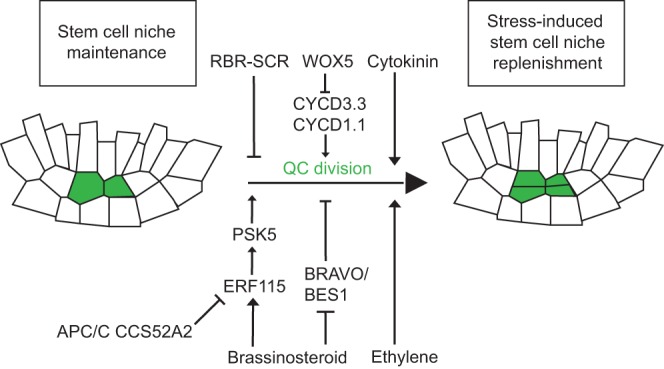
**The molecular control of QC quiescence.** The low cell division rate in the QC is controlled by the interplay between diverse molecular pathways. On the one hand, the QC-specific factor WOX5 directly represses the expression of the cell cycle genes *CYCD1.1* and *CYCD3.3* and thereby inhibits cell division in the QC. RBR also represses QC cell division by forming a protein complex with SCR. On the other hand, the cytokinin, ethylene and brassinosteroid signalling pathways have a positive influence on QC cell divisions. BR activity is twofold: it activates *ERF115* and subsequently *PSK5* but also represses the accumulation of the BRAVO-BES1 complex, which acts as a repressor of QC cell divisions. The active maintenance of this low division rate allows the QC to replenish damaged cells of the stem cell niche upon stress and thereby contributes to stem cell niche longevity.

From the first studies describing the QC (Clowes, 1956) until today, the molecular basis for the low division rate of QC cells has captured great interest, but the underlying mechanisms only now begin to emerge. Only in recent years have a series of genetic and genomic studies shed light on the mechanisms controlling cell quiescence and on its relevance for stem cell niche homeostasis. Interestingly, these studies show that quiescence is required not for stem cell maintenance, but rather to sustain growth upon genotoxic stress, allowing the QC to act as a reservoir for replenishing stem cells after injury (Fig. 3) (Cruz-Ramírez et al., 2013; Heyman et al., 2013; Vilarrasa-Blasi et al., 2014). At the molecular level, it has emerged that RBR cell-autonomously represses asymmetric cell division in the QC, in addition to its role during columella cell differentiation (Wachsman et al., 2011). This repression is dependent on the physical interaction of RBR and SCR and also requires the activity of the transcription factor SHORT ROOT (SHR) (Cruz-Ramírez et al., 2013). Another direct link to cell cycle control is provided by the QC-specific transcription factor WOX5, which controls quiescence by directly repressing the expression of *CYCD1.1* and *CYCD3.3* (Fig. 3) (Forzani et al., 2014).

Interestingly, maintenance of the QC in a slow cycling state also requires a delicate interplay between different phytohormones, such as ethylene, cytokinin and brassinosteroids (BR) (Fig. 3) (Heyman et al., 2013; Ortega-Martinez et al., 2007; Vilarrasa-Blasi et al., 2014; Zhang et al., 2013). Whereas the mechanisms downstream of ethylene and cytokinin still are mostly unresolved, there has been considerable advance in our understanding of BR-mediated control of QC divisions. First, BR promotes the expression of *ETHYLENE RESPONSE FACTOR 115* (*ERF115*), which, in turn, stimulates QC division through transcriptional activation of *PHYTOSULFOKINE PRECURSOR 5* (*PSK5*), which encodes a member of the small peptide phytosulfokine family (Yang et al., 2001). In turn, the activity of ERF115 activity is limited via its proteolysis by the anaphase-promoting complex/cyclosome/CELL CYCLE SWITCH 52 A2 (APC/CS52A2) complex (Heyman et al., 2013). Second, BRs promote QC division by repressing the expression of the MYB transcription factor gene *BRASSINOSTEROIDS AT VASCULAR AND ORGANIZING CENTER* (*BRAVO*), which is required for slow QC divisions. Mechanistically, BR signalling leads to phosphorylation of BRI1-EMS-SUPPRESSOR 1 (BES1), which dimerizes with BRAVO to repress *BRAVO*, thereby constituting a network module with switch-like properties (Fig. 3) (Vilarrasa-Blasi et al., 2014). Although a number of different molecular pathways have been shown to contribute to QC cell quiescence, it remains to be deciphered how they are interconnected to control self-renewal of the QC.

### Linking metabolic state to control of the cell cycle

Homeostatic meristem activity requires a tight control of stem cell proliferation with the developmental and nutritional status of the entire organism. In the vegetative shoot meristem, *WUSCHEL-RELATED HOMEOBOX 9* (*WOX9*) controls stem cell activity by promoting the expression of WUSCHEL. Notably, WOX9 also integrates metabolic signals into stem cell behaviour, as the meristem arrest observed in *wox9* mutant plants can be rescued by promoting cell cycle entry with sucrose (Wu et al., 2005). Along the same lines, a recent study identified the role of photosynthesis in determining the nutritional status that subsequently controls the activity of root meristems (Xiong et al., 2013). This study also delineated a molecular pathway involving a glucose–target-of-rapamycin (TOR) relay in transmitting information on metabolic state from the shoot to the root in order to control cell production in the RAM. Interestingly, the glucose-TOR pathway promotes progenitor cell division independently of *PLT1* and auxin/cytokinin signalling. Instead, TOR kinase activates E2Fa by direct phosphorylation, which in turn promotes its activity and the regulation of a set of S phase genes, ultimately leading to cell cycle progression. This mechanism now clarifies a molecular pathway that translates metabolic state into cellular behaviour to modulate root growth (Xiong et al., 2013).

## The role of chromatin dynamics

From the maintenance of pluripotent stem cells to their commitment to specific lineages, precise gene expression programs need to be deployed and stabilized over an extended period of times. Thus, chromatin-remodelling constitutes an important layer of regulation to stably switch cellular fates during plant and animal development (Lodha et al., 2009). The control of chromatin structure is an ancient cellular function and is shared by many stem cell systems across the kingdoms of life. Consistently, many chromatin-remodelling factors identified in plants were often first discovered in fungal or animal model systems, and display substantial similarities at the structural and functional level (Goodrich et al., 1997; Kaya et al., 2001).

### Chromatin remodelling and meristem organization

**Fig. 4.**
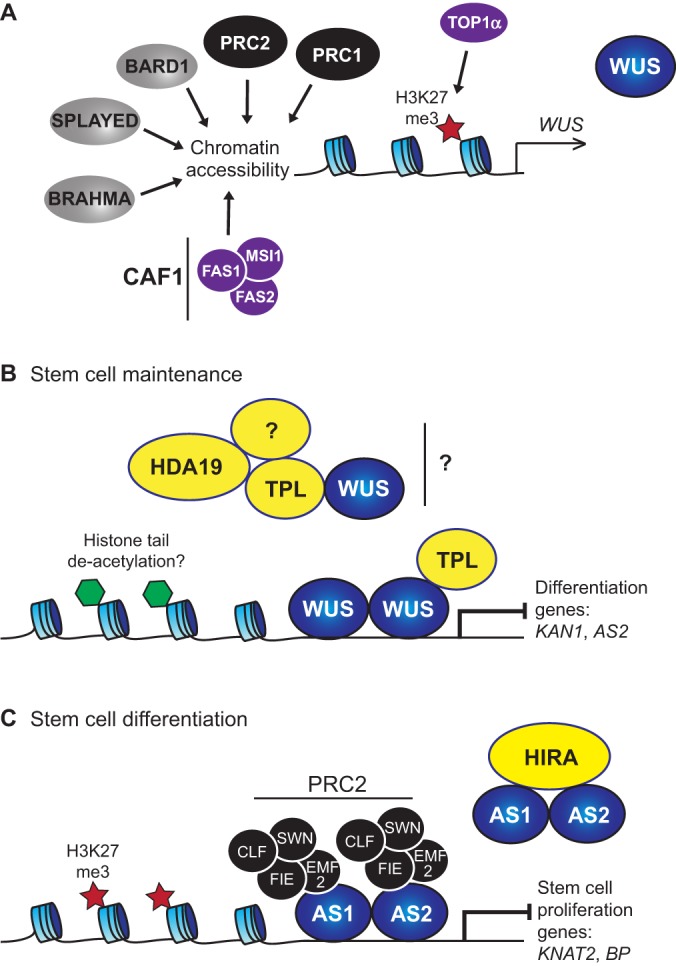
**Chromatin remodelling in meristems.** (A) The expression of the shoot stem cell-inducing factor *WUS* is under the control of many chromatin-remodelling factors. They regulate chromatin accessibility at its regulatory regions and thereby influence stem cell fate maintenance. (B) WUS maintains stem cell fate by directly repressing the expression of differentiation genes (e.g. *KAN1*, *AS2*). WUS forms a homodimer and can also physically interact with TPL, which acts as a transcriptional co-repressor by forming a complex with HDA19 and potentially other factors. Together, WUS, TPL and HDA19 may form a repressive complex by promoting histone tail de-acetylation to exclude the expression of differentiation genes within the SAM. (C) At the transition between peripheral and differentiation zone, an AS1/AS2 complex is crucial for repressing the stem cell proliferation genes *KNAT2* and *BP*. AS1 and AS2 physically interact with the histone chaperone HIRA, but also recruit the PRC2 complex to the promoter of *KNAT2* and *BP* to trigger H3K27me3 and thereby stably repress meristem identity. Blue, transcription factors; yellow, co-repressor complex; purple, DNA replication machinery; black, PcG complex; grey, other DNA-binding factors.

Components of the DNA replication machinery play crucial roles in maintaining chromatin structure through cell division and thus are pivotal for the regulation of pluripotency. Chromatin state is determined in large part by nucleosome occupancy and distribution, with nucleosome assembly at the replication fork carried out by the histone chaperone complex CHROMATIN ASSEMBLY FACTOR 1 (CAF1). Originally identified in human cells, CAF1 is composed of three proteins: p150, p60 and p48. In plants, the CAF1 complex (Fig. 4A) is composed of FASCIATA1 (FAS1), FAS2 and MULTICOPY SUPRESSOR OF IRA1 (MSI1), all of which display high degrees of conservation of their functional domains with their animal counterparts and exhibit a similar histone chaperone activity (Kaya et al., 2001). Loss of FAS function impairs the cellular organization of meristems, and both *WUS* and *SCR* are mis-regulated in *fas* mutant plants (Kaya et al., 2001) (Fig. 4A). Importantly, the post-translational status of histone tails has also a crucial role in modulating chromatin structure and stem cell dynamics (Lodha et al., 2009).

### Chromatin remodelling in the SAM

As *WUS* is the central regulator of stem cell fate in the SAM, it is not surprising that evolution has brought about a highly stabilized regulatory system that controls its expression. The activities of diverse chromatin remodelling factors converge at the *WUS* promoter and thus indirectly influence shoot stem cell activity. TOPOISOMERASE 1-α (TOP1α), which, among other tasks, regulates nucleosome positioning and controls meristem activity (Graf et al., 2010; Liu et al., 2014; Takahashi et al., 2002); *top1α* mutants display a larger SAM, resulting from the initiation of multiple smaller ones, and a reduced root progenitor cell activity (Graf et al., 2010). At the mechanistic level, TOP1α reduces nucleosome occupancy on the promoters of its target genes and facilitates the deposition of histone H3 lysine 27 trimethylation marks at the *WUS* promoter to repress its expression (H3K27me3) (Liu et al., 2014). By contrast, the SWI/SNF-class ATPase protein SPLAYED promotes *WUS* expression by directly binding to its promoter (Fig. 4A) (Kwon et al., 2005). Importantly, other chromatin remodelling factors control SAM integrity, although their specific activity at the regulatory regions of key transcription factors still remains to be demonstrated. Among them, BRAHMA, another member of the SWI/SNF family, is required for correct inflorescence meristem activity (Bezhani et al., 2007; Farrona, 2004). In addition, the DNA repair factor BREAST CANCER ASSOCIATED RING 1 (BARD1) restricts *WUS* expression to the OC (Fig. 4A), and loss of BARD1 function causes dramatic expansion of the stem cell domain together with the formation of large shoot meristem structures (Han et al., 2008). The function of the two PcG complexes is also crucial to restrict *WUS*, as reducing PRC2 activity in the *clf* mutant leads to a substantial increase of *WUS* expression (Barrero et al., 2007). Interestingly, the trxG proteins ULTRAPETALA1 (ULT1) and ULT2 are also involved in controlling inflorescence meristem activity, and *ult* loss-of-function mutants develop larger shoot meristems (Fletcher, 2001; Monfared et al., 2013).

The regulation of chromatin state is not only important to tune *WUS* expression, but might also play a significant role in translating WUS function into appropriate cell behaviour via its target genes. Within the SAM, WUS mainly acts as a transcriptional repressor and prevents the expression of SAM differentiation genes (Fig. 4B) (Busch et al., 2010; Ikeda et al., 2009; Yadav et al., 2013). The observations that WUS physically interacts with the Gro/Tup1 co-repressor TOPLESS (TPL) and that fusing TPL to a non functional version of WUS protein partially rescues WUS function suggest that WUS is part of a repressor complex involving TPL. Interestingly, TPL in turn is able to interact with HISTONE DEACETYLASE 19 (HDA19), which promotes histone de-acetylation (Fig. 4B) and seems to be required for the activity of the APETALA2 (AP2) transcription factor in flowers (Causier et al., 2012; Gonzalez et al., 2007; Krogan et al., 2012). However, it still remains unclear whether the connection between WUS and chromatin remodelling through histone de-acetylation plays a role for shoot stem cell pluripotency.

Whereas chromatin states in the centre of the SAM are stabilized in order to maintain stem cell identity, cell proliferation factors such as members of the KNOX gene family need to be stably repressed outside the SAM (Fig. 4C) (Byrne et al., 2000). At the shoot meristem-organ boundary, the formation of AS1-AS2 dimers is required for this fate switch, as they directly repress the expression *KNAT2* and *BP* (Guo et al., 2008; Lodha et al., 2013). Mechanistically, AS1-AS2 dimers recruit HIRA proteins and members of the PRC2 complex to the promoters of *KNAT2* and *BP*, thereby stimulating H3K27me3 deposition and reducing the occurrence of the active H3K4me3 mark (Fig. 4C). This in turn promotes binding of the PRC1 complex factor LIKE HETEROCHROMATIN PROTEIN 1 (LHP1), which allows further chromatin compaction, stable repression of gene expression and ultimately triggers a cell fate switch that persists during organogenesis (Guo et al., 2008; Lodha et al., 2013; Phelps-Durr et al., 2005).

### Chromatin remodelling and root cell fate

The dynamic regulation of chromatin organization is also intimately involved in specifying cellular fates in the root. By analysing histone mobility in the RAM, Rosa et al. demonstrated that as RAM cells progress from the transit-amplifying zone to the differentiation zone, histone mobility decreases, which correlates with a reduction of their acetylation. Furthermore, histone mobility in root stem cells appears to be lower than in the surrounding cells, suggesting that flexible chromatin organization might be important for stem cell fate (Rosa et al., 2014). Modulation of chromatin state is also important for hair/non-hair cell patterning, which is dependent on the relative position of epidermal cells to the underlying cortical cell layer (Berger et al., 1998). Whereas this developmental switch does not directly influence cellular pluripotency, the underlying regulatory system is an excellent example of how positional information is translated into stable cell identities in plants. The GLABRA2 (GL2) transcription factor, expressed exclusively in non-hair cells, acts in neighbouring cells to induce hair cell fate (Masucci et al., 1996). An elegant study demonstrated that chromatin at the GL2 locus is in an open state in non-hair cells, whereas it is more compact in hair cells (Costa and Shaw, 2006). Importantly, chromatin state is not cell lineage-dependent, but dynamically established in response to cell position. This process requires the activity of the histone chaperone complex CAF1, but also of GL2-EXPRESSION MODULATOR (GEM), which influences the H3K9me2/3 methylation status (Caro et al., 2007). Thus, the stereotypical regulation of root hair cell fate is a prominent example of how positional information acts upstream of chromatin remodelling in order to ultimately instruct diverse genetic programs leading to cell fate commitment.

## Reprogramming and cellular plasticity

Plant stem cell niches are highly dynamic environments that have to integrate environmental signals and to respond accordingly to sustain growth. As stem cells become committed towards a particular cell lineage in response to positional information, they will adopt a specific fate and behaviour. Interestingly, perturbation studies reveal that stem cell systems display an important flexibility, in which cellular properties can be reprogrammed to fit the overall stability of the tissue. This process includes the activity of key regulators that control stem cell niche homeostasis.

### Reprogramming cellular fate in plants after injury

Experiments using laser ablations to challenge stem cell systems have provided exciting insights into the mechanisms underlying plant cell pluripotency and developmental plasticity (Reinhardt et al., 2003; van den Berg et al., 1997, 1995). In the SAM, ablation of the entire stem cell domain and OC triggers the *de novo* formation of a stem cell system from surrounding cells, which quickly regenerates a functional shoot meristem (Reinhardt et al., 2003). Interestingly, only one day after removal of the OC and CZ, cells at the periphery are reprogrammed and start expressing *WUS* (Reinhardt et al., 2003). In the root, similar types of experiments were performed by successively ablating root cortical initials or their daughter cells. These assays revealed that cells pushed into the position of the ablated cell by their neighbours lose their original identity and acquire the cell fate of the ablated cell. These experiments clearly demonstrated the plasticity of plant cell fate *in vivo* and demonstrated the dominating role of positional information for fate acquisition (van den Berg et al., 1995). Furthermore, ablating the QC revealed that these cells act non-cell autonomously to maintain stem cell fate of the surrounding initials (van den Berg et al., 1997). By analysing a whole suite of molecular reporters, it was also shown that a new functional QC is established three days after ablation. This regeneration first involves the modification of auxin flux, thereby inducing the expression of the stem cell maintenance factors *SCR*, *PLT* and *SHR*, which subsequently reorganize and reinforce auxin transport and signalling (Xu et al., 2006). The regenerative capacity of the RAM was further investigated by root tip removal experiments, which showed that the stem cell niche is able to regenerate even after removing 200 µm of the root tip. Interestingly, this process is independent of the activity of PLT, SCR or SHR, which indicates that the regeneration process does not require a functional stem cell system (Sena et al., 2009). Importantly, as in the QC ablation experiments, auxin transport and distribution are crucial to instruct this regeneration process and seem to act upstream of the stem cell identity genes to specify *de novo* meristematic tissues displaying self-organizing properties (Sena et al., 2009). This raises important questions regarding the molecular control and the sequence of cellular events acting downstream of auxin signalling during cell fate re-specification and tissue regeneration.

### Induced pluripotency in plants?

The reprogramming of animal somatic cells into embryonic-like stem cells (induced pluripotent stem cells; iPSCs) highlights the fundamental capacity of many cell types to re-enter pluripotency from a differentiated state. Strikingly, the concerted activity of only four transcription factors (OCT4, SOX2, KLF-4 and c-Myc) is sufficient to initiate the molecular reprogramming of animal somatic cells to a pluripotent state *in vitro* (Takahashi and Yamanaka, 2006). Whereas the *in vitro* reprogramming of plant tissue was achieved more than 50 years ago using simple phytohormone treatments (see Box 2), more recent studies have shown that reprogramming to a pluripotent state is even possible *in vivo* for a limited subset of cell types: the ectopic expression of *STM* or the co-expression of *STM* with *WUS* in the hypocotyl is sufficient to induce the formation of meristem-like structures (Brand, 2002; Gallois et al., 2002; Lenhard et al., 2002; Scofield et al., 2013). Furthermore, mis-expressing *WUS* in the root, where it is normally absent, promotes ectopic formation of shoot structures (Gallois et al., 2004). Conversely, expression of *PLT2* in shoot tissues leads to the development of roots from aerial tissues (Galinha et al., 2007). Collectively, these experiments demonstrate that plant cell reprogramming can be achieved *in vivo* using the ectopic expression of a small number of key transcriptional regulators (see Box 1).

Box 1. **Pluripotency, multipotency, transdifferentiation and reprogramming in plants**

The term pluripotency has been defined as the ability of stem cells to give rise to all three animal germ layers (endoderm, mesoderm and ectoderm). It was originally used to describe the property of embryonic stem cells derived from the inner cell mass of mammalian embryos, which, after culture under appropriate conditions, can generate all cell types constituting the animal body. As stem cells progress towards a fixed lineage, their fate becomes more restricted to give rise to a limited subset of cell types (multipotent) before they finally differentiate.

A parallel definition is currently used for plant stem cells located in meristems, as they are able to give rise to all cell types of shoot or root, respectively. Whereas this behaviour is more comparable to multipotency in animals, mutations or ectopic expression of transcriptional regulators are able to overcome this fate limitation *in vivo*, suggesting that meristematic cells could indeed be pluripotent.

In another analogy to animal cells, excised plant tissue is able to produce undifferentiated proliferating cells, termed callus, under appropriate phytohormone treatment *in vitro*. These cells then can differentiate into a fully functional plant, supporting the idea that many plant cells are pluripotent. However, recent work suggests that callus cells are derived from a small number of stem cell-like cells distributed in all plant tissues, rather than a re-programming of terminally differentiated cells.

Thus, whereas the terminology derived from classical studies in animals might not perfectly fit the behaviour of plant stem cells, it still seems a valid concept. In addition, the use of shared terminology greatly facilitates communication between researchers studying both kingdoms.

Box 2. **Historical perspectives on plant stem cell research**

**1759:** First description of the shoot apical meristem by Caspar Wolff

**1926:** Characterization of the phytohormone auxin by Frits Warmolt Went

**1939:** Philip White develops the first plant tissue culture

**1955:** First isolation of the cytokinin kinetin by Miller and colleagues

**1957:** Controlled organogenesis from callus by Skoog and Miller

**1964-1965:** Regeneration of complete plant from single isolated carrot and tobacco cultured cells by Vasil and Hildebrandt

**1991:** Cloning of the shoot meristem maintenance gene KNOTTED1 by Hake and colleagues

**1993:** First description of the *Arabidopsis thaliana* RAM cellular organization by the Scheres lab

**1995; 2003:** Ablation studies by the Scheres and Kuhlemeier labs show self-organization properties of plant stem cell systems and strict dependence of cell fate on cellular position

**1998:** Cloning of shoot stem cell maintenance gene *WUSCHEL* by Laux and colleagues

**2000:** Description of the WUS/CLV3 feedback loop of communication by the Laux, Simon and Meyerowitz labs

**2005; 2008; 2010:** Mechanisms of phytohormonal cross-talk underlying stem cell activity identified by the Sabatini and Lohmann labs

**2010:** Identification of distributed callus founder cells in all plant tissues that promote *in vitro* proliferation and differentiation via a lateral root developmental program by the Meyerowitz lab

Plant tissue culture techniques, which allow the somatic propagation of individual plants, also provide interesting insights into the regulation of pluripotency. Classical callus induction experiments demonstrated the capacity of isolated differentiated plant tissues to regenerate full adult organisms. Notably, the molecular events and players involved during callus induction and shoot regeneration closely mirror those involved in the control of stem cell systems *in vivo*. For example, auxin and cytokinin signalling are crucial for this process, and shoot regeneration is impaired in *wus* and *pin1* mutant plants (Gordon et al., 2007). Conversely, callus formation can be uncoupled from cytokinin signalling in tissues ectopically expressing WUS or in plants lacking negative feedback regulators of cytokinin, which are repressed by WUS *in vivo* (Buechel et al., 2010; Zuo et al., 2002).

Although all plant tissues can potentially form callus, the question as to whether all plant cell types can be reprogrammed to a pluripotent state has been a matter of debate (Sugimoto et al., 2011). Shoot regeneration from root explants revealed that, upon callus induction, xylem-pole pericycle (XPP) cells start to proliferate and form cell masses. Importantly, callus formation and shoot regeneration are severely impaired upon ablation of these XPP cells (Che et al., 2007). In line with these observations, callus formation follows a lateral root-like molecular program independently of the origin of the tissue. Furthermore, interfering with *ABERRANT LATERAL ROOT FORMATION 4* (*ALF4*) function, which controls lateral root development, impairs callus formation (Sugimoto et al., 2010). These findings suggest that callus formation is not the result of reprogramming somatic cells towards pluripotency but rather is caused by the expansion of a pre-existing stem cell-like population (Sugimoto et al., 2010). Further investigations are therefore required to fully clarify the role of differentiated plant cells during tissue reprogramming towards a pluripotent state.

## Conclusion and outlook

Following the detailed description of the molecular mechanisms controlling plant stem cell pluripotency, one might wonder about the relevance of such an elaborate system for plant survival. Against this backdrop, we have to consider that, owing to their sessile mode of life, plants are not only subjected to substantial fluctuations in their direct abiotic environment but are also threatened by herbivores and pathogenic microbes. Consequently, plant development must be robust, yet plastic in face of these challenges, and pools of continuously active pluripotent stem cells obviously represent an ideal cellular toolkit for this kind of lifestyle. However, there is more to it, as even when stem cell systems are ablated physically or genetically, regeneration can take place, suggesting that other cells can acquire stem cell properties, similar to what is observed in a wide range of animal stem cell systems (Krieger and Simons, 2015). This dynamic behaviour results from regulatory interactions at various levels. First, at the molecular level, pluripotency results from an elaborate interplay between phytohormonal signalling, key transcription factors and chromatin remodelling. Importantly, phytohormones exhibit substantial spatio-temporal dynamics, which are translated by key transcriptional regulators and, with the contribution of chromatin remodelling, result in stable gene expression programs and thus tissue organization. It will be imperative to further decode the mechanisms by which environmental and metabolic signals impinge on this regulatory program and to understand how this is translated into cell behaviour. Second, at the cellular level, plant tissues carry dispersed cells that are able to quickly enter a pluripotency program and to proliferate, resulting in the production of the full complement of plant cell types. In this context, more research is required to elucidate the nature and position of these cells and to delineate the signals leading to their activation. Third, at the tissue level, positional information plays a dominant role in plant cell fate acquisition and control of the cell cycle, giving rise to a highly plastic developmental program. Again, our appreciation of the signals encoding this spatio-temporal information is still limited and requires further refinement.

A final, important aspect to consider is the high degree of evolutionary conservation of the pluripotency network within the plant kingdom. This not only includes sequence conservation of important regulators, such as the WOX genes, between species, but also extends to the cellular and molecular design of the diverse stem cell systems within a given species. The fact that plant stem cell regulation largely relies on multi-purpose building blocks, such as the CLE-WOX-HAM module or the cytokinin signalling system, not only promises that discoveries made in reference plant species will be applicable to crop plants, but also that the experimental strategies limited to individual stem cell systems can inform us about the general mechanisms underlying the control of pluripotency in multiple stem cell niches.
